# TNFα promotes oral cancer growth, pain, and Schwann cell activation

**DOI:** 10.1038/s41598-021-81500-4

**Published:** 2021-01-19

**Authors:** Elizabeth Salvo, Nguyen H. Tu, Nicole N. Scheff, Zinaida A. Dubeykovskaya, Shruti A. Chavan, Bradley E. Aouizerat, Yi Ye

**Affiliations:** 1 Bluestone Center for Clinical Research, New York University College of Dentistry, 421 First Avenue, 233W, New York, NY 10010 USA; 2 Department of Oral Maxillofacial Surgery, New York University College of Dentistry, New York, USA; 3 Department of Molecular Pathobiology, New York University College of Dentistry, New York, USA; 4 Department of Neurobiology, School of Medicine, University of Pittsburgh, Pittsburgh, PA USA; 5 Graduate School of Arts and Science, Department of Biology, New York University, New York, USA

**Keywords:** Cancer, Neuroscience

## Abstract

Oral cancer is very painful and impairs a patient’s ability to eat, talk, and drink. Mediators secreted from oral cancer can excite and sensitize sensory neurons inducing pain. Cancer mediators can also activate Schwann cells, the peripheral glia that regulates neuronal function and repair. The contribution of Schwann cells to oral cancer pain is unclear. We hypothesize that the oral cancer mediator TNFα activates Schwann cells, which further promotes cancer progression and pain. We demonstrate that TNFα is overexpressed in human oral cancer tissues and correlates with increased self-reported pain in patients. Antagonizing TNFα reduces oral cancer proliferation, cytokine production, and nociception in mice with oral cancer. Oral cancer or TNFα alone increases Schwann cell activation (measured by Schwann cell proliferation, migration, and activation markers), which can be inhibited by neutralizing TNFα. Cancer- or TNFα-activated Schwann cells release pro-nociceptive mediators such as TNFα and nerve growth factor (NGF). Activated Schwann cells induce nociceptive behaviors in mice, which is alleviated by blocking TNFα. Our study suggests that TNFα promotes cancer proliferation, progression, and nociception at least partially by activating Schwann cells. Inhibiting TNFα or Schwann cell activation might serve as therapeutic approaches for the treatment of oral cancer and associated pain.

## Introduction

Oral cancer features higher pain prevalence and intensity than other cancer type^1,2^. Patients with oral cancer rate pain as the worst symptom, as they suffer from severe, chronic, mechanically induced pain^3,4^. Oral cancer pain impairs a patient’s speech, eating, drinking, and interpersonal relations^5^. While opioids, the gold standard therapy, may provide some pain relief initially, they are associated with undesired side effects^6^. There are no alternative analgesic regimens available for intractable cancer pain once patients develop tolerance to opioids.

Oral cancers produce and secrete algogenic mediators that activate and sensitize primary afferent neurons to initiate pain^7–9^. One such mediator is TNFα, a “master regulator” cytokine that initiates inflammation and drives pro-inflammatory cytokine cascades^10^. Many cytokines downstream of TNFα, such as nerve growth factor (NGF) and interleukin 6 (IL-6), have been implicated in oral cancer pain^8,9,11^. We found that TNFα is secreted from oral cancer cell lines and upregulated in cancer tongue tissues collected from mice treated with the carcinogen 4-nitroquinoline 1-oxide (4NQO)^9^. Inhibiting TNFα signaling abolished oral cancer-evoked functional allodynia and disrupted T cell infiltration in mice^9^. The analgesic effect of TNFɑ inhibitors in animal models of oral cancer has yet to be determined. Clinically, the role of TNFɑ in oral cancer pain in patients remains unknown.

Schwann cells, the peripheral glia that ensheathes peripheral nerves, may be a major source for pro-inflammatory mediators that contribute to cancer growth and pain. During nerve injury, the quiescent Schwann cells transform into an activated form, become proliferative and migratory, and release pro-inflammatory mediators such as TNFα and NGF^12^. Similar to their response to nerve injury, Schwann cells are activated by the presence of cancer cells or hypoxia, an oxygen deficient environment common in cancer^11,13–15^. Activated Schwann cells have been shown to promote cancer proliferation, dispersion, invasion, and metastasis in cancers of the skin, pancreas, colon, lung, and head and neck^11,13,16–19^.

The objectives of the present study are to determine whether TNFα (1) contributes to oral cancer pain in patients and animals with oral cancer; and (2) activates Schwann cells resulting in oral cancer progression and pain. We measured TNFα in human oral cancer tissues and correlated TNFα concentration with reported pain scores in patients. We examined the effect of TNFα inhibition on cancer growth, Schwann cell activation, and pain-like behaviors using cell culture and animal models of oral cancer.

## Results

### TNFα concentration in oral cancer tissues correlates with pain scores in patients

**Figure 1:**
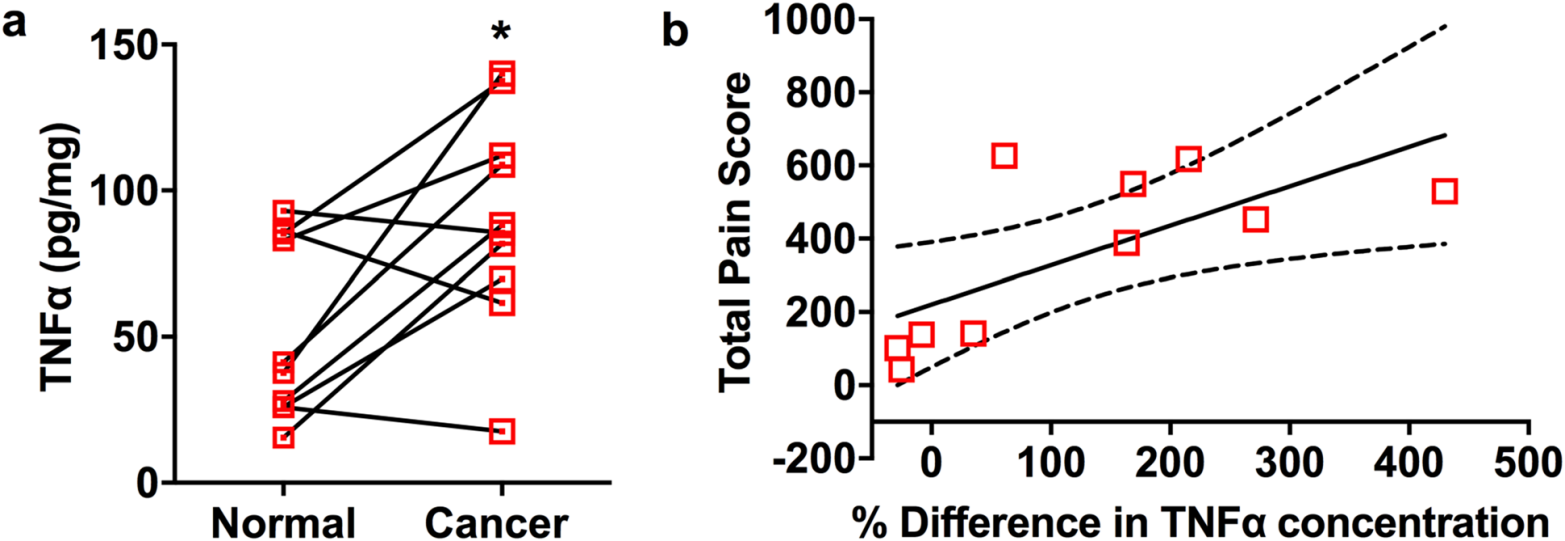
TNFα is correlated with human oral cancer pain scores. (**a**) TNFα protein concentration is higher in cancer tissues compared to anatomically matched contralateral healthy tissues from the same patient (n = 10, *P < 0.05, paired t-test). (**b**) Patients were asked to answer the Oral Cancer Pain Questionnaire before surgery. The mean pain score from patients correlated positively with percentage change in TNFα concentration between cancer and matched contralateral normal tissues (*r* = 0.7, *P* < 0.05).

To examine whether TNFα released into the cancer microenvironment is associated with oral cancer pain in humans, we assessed oral cancer pain using a validated oral cancer pain questionnaire^20,21^ and measured TNFα protein concentration in resected oral tumors in patients. We also quantified TNFα protein concentration from anatomically matched healthy tissues collected from each patient to control for individual variations in TNFα protein concentration at the basal level. The TNFα concentration in resected oral cancer tissues was significantly higher than anatomically matched healthy tissues from the same patients (Fig. 1a). Mean total pain scores reported by patients correlated positively with the percentage change of TNFα concentration between the cancer and the matched contralateral normal tissues (*r* = 0.7, *P* < 0.05, Fig. 1b).

### TNFα antagonism inhibits nociception in mice with cancer

**Figure 2:**
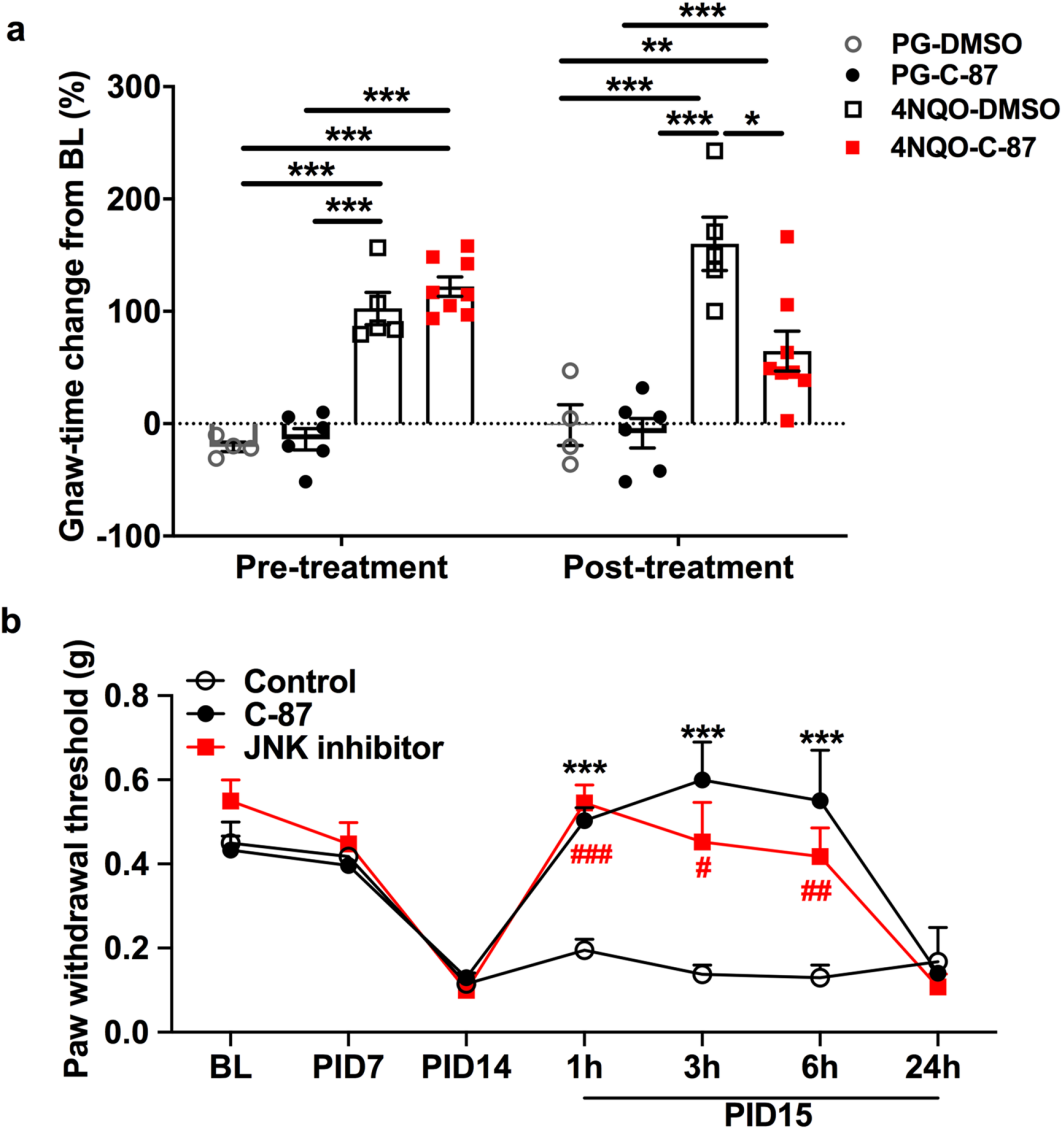
Blocking TNFα or JNK inhibits nociception in mice with cancer. (**a**) After 16 weeks of 4NQO treatment, mice exhibited significant increase in gnaw-time from its respective baseline (pre-injection). Propylene glycol (PG) treatment did not affect gnaw-time. In 4NQO tongue cancer mice, C-87 (12.5 mg/kg) IP injection significantly reduced percentage of gnaw-time change from baseline (n = 8) 1 h post-injection than the vehicle (10% DMSO) treated cancer mice (n = 5). C-87 (n = 6) or vehicle (n = 4) had no effect in non-cancer mice treated with PG alone. (**b**) Mice with paw SCC developed cancer pain at PID7. C-87 and the JNK inhibitor SP600125 treatment significantly reduced mechanical nociception compared to vehicle at 1, 3, and 6 h after treatment compared to the control group. 24 h after the treatment the analgesic effect of C-87 was gone (n = 5 per group, Two-way ANOVA). *P < 0.05; **P < 0.01; ***P < 0.001.

To investigate the therapeutic effect of TNFα antagonism in cancer pain we used two cancer models, treated the animals with a TNFα neutralizing compound C-87^22^, and measured nociceptive behavior. We produced a 4NQO oral carcinogenesis model that is anatomically relevant to human oral cancer^9,23^. Oral nociception was quantified using a validated gnawing assay; increased gnaw-time from baseline is indicative of increased nociception^24^. Mice treated with 4NQO in the drinking water for 16 weeks exhibited a significant increase in gnaw-time from baseline (Fig. 2a). Water containing propylene glycol alone (vehicle control) had no effect on the mouse gnaw-time. C-87 injected (12.5 mg/kg, n = 6) 4NQO cancer mice exhibited a reduced gnaw-time increase from baseline compared to vehicle injected (10% DMSO) 4NQO cancer mice (*P* < 0.001, n = 5, Fig. 2a). C-87 (n = 6) or DMSO (n = 4) injection did not affect gnaw-time in mice who received propylene glycol alone. To confirm the anti-nociceptive effect of TNFα antagonism, we next used a paw xenograft model that allows the use of the paw withdrawal assay, the gold standard for the assessment of mechanical allodynia in rodents^25^. The paw cancer model is generated by inoculating HSC-3 cells, a human oral squamous cell carcinoma (SCC) cell line, into the mouse right hind paw^7,8,26–28^. At post-inoculation day (PID) 14, mice with paw tumors exhibited reduced paw withdrawal threshold compared to baseline (Fig. 2b). Tumor-bearing mice treated with C-87 (12.5 mg/kg, n = 5) demonstrated increased paw withdrawal thresholds from 1 h up to 6 h after treatment compared to the vehicle group (n = 5, *P* < 0.001, Fig. 2b). Since TNFα is known to activate the c-Jun N-terminal kinase (JNK) to cause persistent pain, we measured the analgesic effect of JNK inhibitor, SP600125, on oral cancer-induced mechanical allodynia in the hindpaw. JNK inhibitor (30 mg/kg, n = 5) increased paw withdrawal thresholds from 1 h up to 6 h after treatment compared to the vehicle group (n = 5, *P* < 0.001, Fig. 2b). The analgesic effect of both C-87 and SP600125 was lost at 24 h following injection in mice with paw tumors.

### TNFα antagonism reduces oral cancer progression and cytokine release

**Figure 3:**
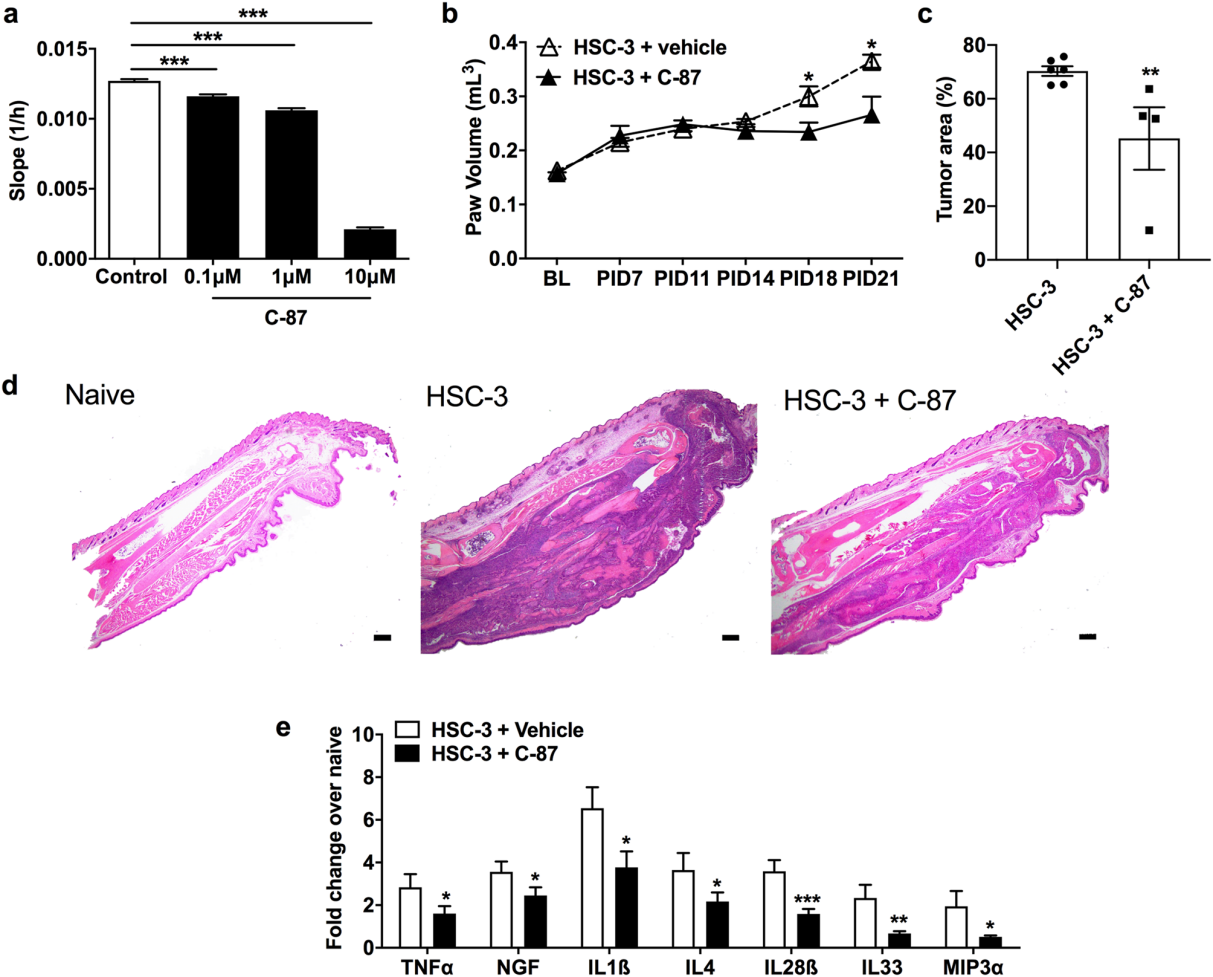
Blocking TNFα inhibits cancer cell growth, migration, and cytokine release. (**a**) Growth rate, measured with the RTCA, following different doses of C-87 treatment in HSC-3 cell culture. C-87 inhibited oral cancer cell growth in a dose dependent manner. One-way ANOVA with Tukey's post hoc analysis. (**b**) Mice with C-87 treatment (n = 7) exhibited a significant decrease in the paw volume compared to the vehicle control mice (n = 6) at PID14, 18, and 21 (two-way ANOVA). Arrow indicates C-87 injection. (**c**) C-87 treated paw cancer mice (n = 6) had smaller tumor area relative to the total paw area compared to vehicle treated paw cancer mice (n = 4). Tumor areas and total paw areas were quantified using H&E stained paw sections. Mann–Whitney U-test. (**d**) Representative H&E stained pictures showing a normal mouse paw, a cancer mouse paw, and a cancer paw treated with C-87 (10 × inset). Scale bar: 100 μm. Images were taken and quantified using Nikon imaging software NIS-Elements F Ver4.60.00. (**e**) C-87 treatment reduced the concentration of TNFα, NGF, IL1β, IL4, MIP3α, IL28β, and IL33 in the paw tumor. Data were presented as fold change of cytokines/chemokines measured from tumor paws over normal paws. n = 6 per group. Mann–Whitney U test. *P < 0.05; **P < 0.01; ***P < 0.001.

Previous reports have indicated a pro-tumorigenic role for TNFα in oral cancer^29–34^. We examined the effect of TNFα antagonism on oral cancer growth in vitro and in vivo. Using the real-time cell analyzer (RTCA) that measures cell resistance as an indicator of cell proliferation^11,35^, we found that C-87 reduced HSC-3 cell growth in a concentration dependent manner compared to the control (*P* < 0.01 at 100 nM and *P* < 0.001 at 1 μM and 10 μM, Fig. 3a). In the paw xenograft SCC model, mice treated with C-87 exhibited smaller paw volume compared to the vehicle-treated tumor-bearing mice at PID18 and PID21 (*P* < 0.05, Fig. 3b). Furthermore, using hematoxylin and eosin (H&E) stained sections of the paw, we found that the percentage of tumor area relative to the total paw area was smaller in C-87 treated mice than the vehicle control (*P* < 0.01, Fig. 3c,d). As inflammation is known to increase oral cancer progression^30^, we used a MILLIPLEX MAP magnetic bead immunoassay to measured pro-inflammatory cytokines in mouse tumor tissues following C-87 treatment. Mice treated with C-87 exhibited lower concentrations of the following pro-inflammatory cytokines in the tumor paw compared to vehicle-treated tumor-bearing mice: TNFα (p < 0.05), NGF (*P* < 0.05), IL1β (*P* < 0.05), IL4 (*P* < 0.05), IL28β (*P* < 0.001), IL33 (*P* < 0.01), MIP3α (*P* < 0.01) (Fig. 3e). The other 35 cytokines measured from the MILLIPLEX MAP magnetic bead immunoassay were not significantly affected by the C-87 treatment (data not shown).

### TNFα antagonism disrupts oral cancer induced Schwann cell proliferation and mutual attraction between Schwann cells and oral cancer cells

**Figure 4:**
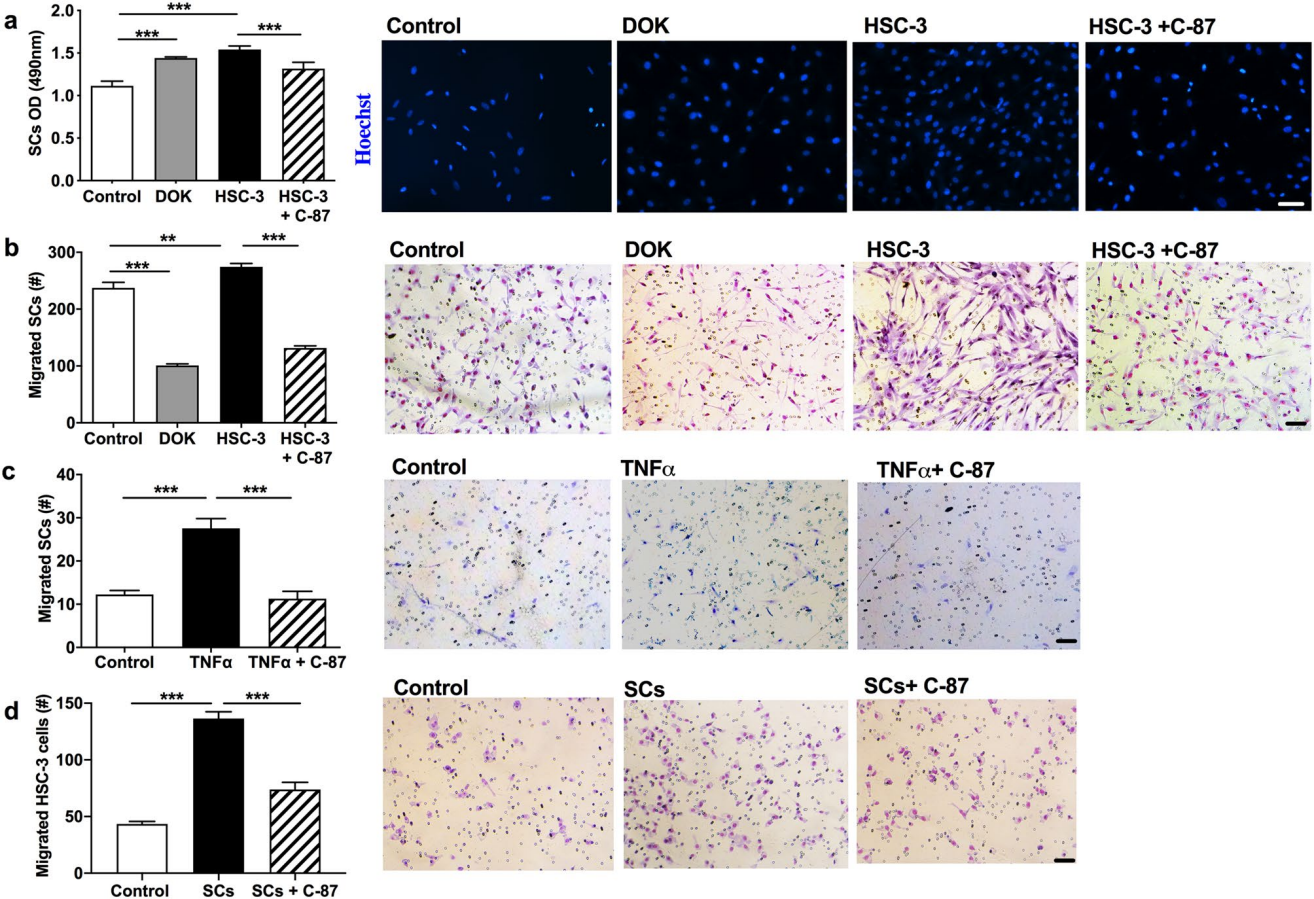
TNFα mediates Schwann cell proliferation and migration in vitro. (**a**) The presence of either DOK or HSC-3 cells in cell inserts increased Schwann cell proliferation 48 h following co-culture in the MTS assay. Increased Schwann cell proliferation induced by the presence of HSC-3 cells was inhibited by adding C-87 into inserts. Representative images of cells with Hoechst stain were shown under each culture condition. OD: optical density. (**b**) Schwann cells are more migratory in the presence of HSC-3 cells compared to the DMEM control while DOK reduced Schwann cell migration. Adding C-87 into the HSC-3 culture reduced Schwann cell migration. (**c**) Adding TNFα to the media at the bottom chamber increased Schwann cells migration compared to the DMEM control. Neutralizing TNFα with C-87 decreased Schwann cell migration. (**d**) Schwann cells induced increased HSC-3 cell migration compared to the DMEM control; adding C-87 (20 µM) into the Schwann cell culture in the bottom chamber blocked this increase. (**b**–**d**), images shown are representative diff-quick stained migrated cells. a-d, one-way ANOVA with Tukey's post hoc analysis. SCs: Schwann cells. Scale bar: 100 μm. *P < 0.05; **P < 0.01; ***P < 0.001. Images were taken using Nikon imaging software NIS-Elements F Ver4.60.00.

We have previously demonstrated that rat Schwann cells (RSC-96) and human oral SCC cells (HSC-3) reciprocally interact to promote proliferation, migration, and invasion^11^. Here we found that human Schwann cells increased migration and proliferation in the presence of human oral cancer cells as well; the growth rate of human Schwann cells was increased in the presence of either precancer dysplastic oral keratinocytes (DOK) or HSC-3 cells grown in culture inserts (*P* < 0.001, Fig. 4a). Adding C-87 (20 μM) to the inserts containing HSC-3 cell culture reduced Schwann cell proliferation (*P* < 0.001, HSC-3 versus HSC-3 + C-87, Fig. 4a). Furthermore, HSC-3 cells stimulated Schwann cells migration compared to the control (Dulbecco's Modified Eagle Medium (DMEM), *P* < 0.01, Fig. 4b), and this increased Schwann cell migration towards HSC-3 cells was inhibited by adding C-87 into the HSC-3 cell culture (*P* < 0.01, DMEM control vs. HSC-3 cells; *P* < 0.001, HSC-3 cells vs. HSC-3 + C-87, Fig. 4b). Recombinant TNFα (20 ng/ml) induced increased Schwann cell migration compared to the DMEM control (*P* < 0.001), which was reversed by adding C-87 into the bottom chamber containing TNFα (*P* < 0.001, Fig. 4c). HSC-3 cells increased their migration towards Schwann cells compared to DMEM control (*P* < 0.001). Adding C-87 into the Schwann cell culture reduced HSC-3 cell migration towards Schwann cells (*P* < 0.001, Fig. 4d).

### *TNFα upregulates c-Jun, GFAP, p75 but downregulates MBP in Schwann cells *in vitro

**Figure 5:**
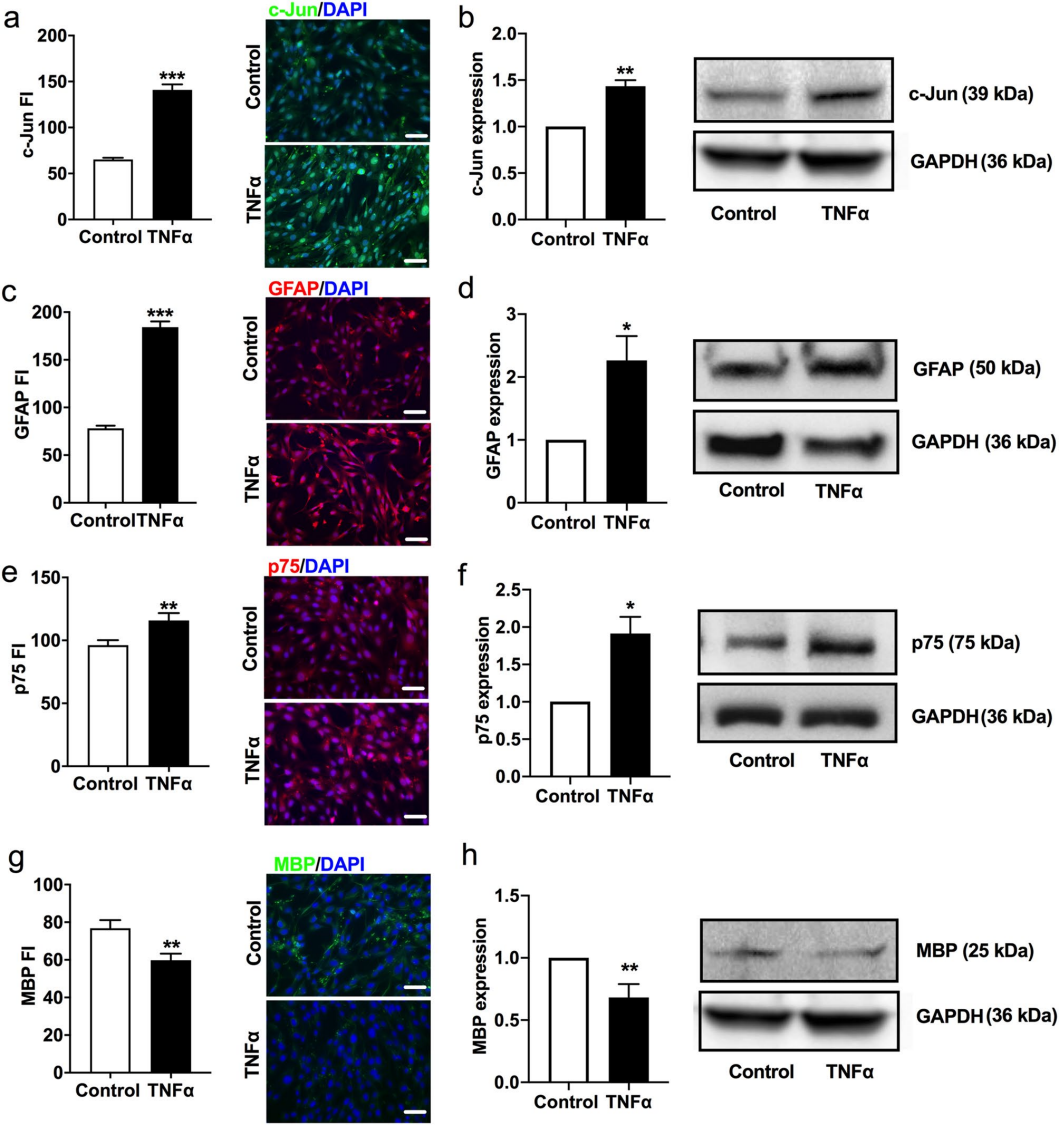
The effect TNFα on the expression of Schwann cell activation markers in vitro. TNFα treatment increased c-Jun (**a**,**b**), GFAP (**c**,**d**), and p75 (**e**–**f**) immunofluorescence intensity and protein expression in cultured Schwann cells compared to the DMEM control. TNFα treatment decreased MBP immunofluorescence intensity and protein expression in cultured Schwann cells compared to the DMEM control (**g**–**h**). Full-length gel blots were provided in the Supplemental Fig. S1 online. Scale bar: 100 μm. Student’s t-test. *P < 0.05; **P < 0.01; ***P < 0.001.

**Figure 6:**
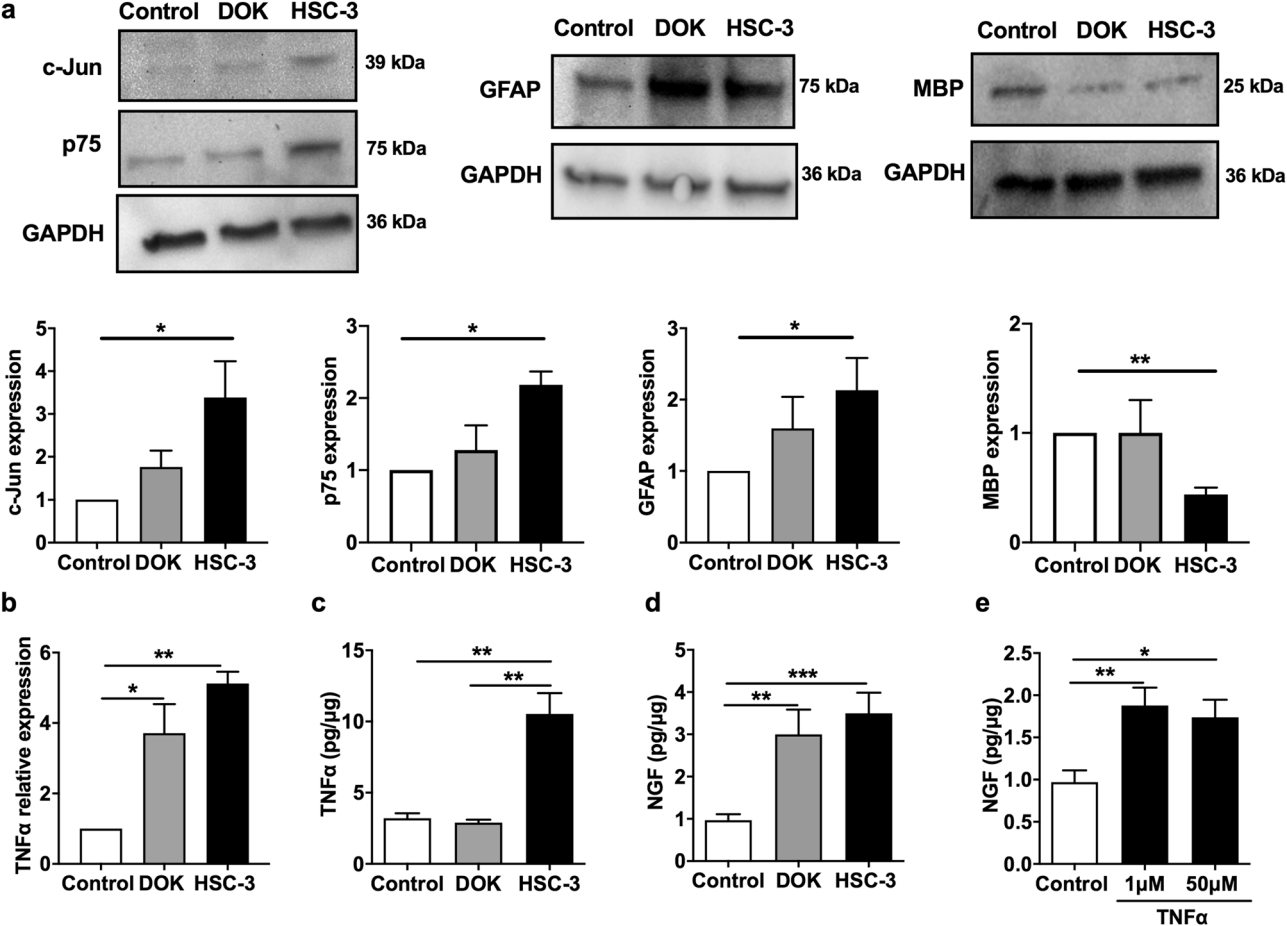
Activated Schwann cells release increased NGF and TNFα. (**a**) Schwann cells co-cultured with HSC-3 cells overexpressed c-Jun, GFAP, p75 but downregulated MBP compared to control Schwann cells (media alone). Full-length gel blots were provided in Supplemental Fig. S2 online. (**b**) Both DOK and HSC-3 co-cultures increased TNFα mRNA expression in Schwann cells compared to control Schwann cells. (**c**) TNFα protein concentration in Schwann cells co-cultured with either DOK or HSC-3 cells compared to control Schwann cells. (**d**) HSC-3 cell or DRK co-culture increased NGF release in Schwann cells compared with control Schwann cells. (**e**) Adding TNFα in cell culture media stimulated increased NGF release compared with control Schwann cells. One-way ANOVA. *P < 0.05; **P < 0.01; ***P < 0.001.

Nerve injury is associated with activated Schwann cells which overexpress c-Jun, p75^NTR^, and GFAP as well as downregulate myelin basic protein (MBP)^14,15,36^. To determine whether TNFα activates Schwann cells to produce a similar phenotype, we stimulated Schwann cells with TNFα in culture and measured Schwann cell activation markers, c-Jun, p75^NTR^, GFAP, and MBP. Schwann cells treated with TNFα (20 ng/ml) showed increased protein expression of c-Jun (Fig. 5a,b), GFAP (Fig. 5c,d), and p75^NTR^ (Fig. 5e,f) compared with the control cells (grown in DMEM alone). MBP protein expression was decreased in Schwann cells treated with TNFα compared to control cells (Fig. 5g,h). Co-culture of Schwann cell with HSC-3 cancer cells also resulted in Schwann cell activation as confirmed by the overexpression of c-Jun, GFAP, p75^NTR^, and downregulation of MBP (Fig. 6a).

### Cancer- or TNFα-activated Schwann cells release TNFα and NGF

We sought to determine the contribution of cancer-activated Schwann cells to oral cancer pain. Pro-inflammatory mediators TNFα and NGF have been highly implicated in cancer pain^8,9,37^; however, the source of these mediators in unclear. Furthermore, a positive feedback loop between NGF and TNFα has been reported in the central glia activation; TNFα can induce NGF expression and vice versa^38^. We measured TNFα and NGF release from Schwann cells following co-culture with HSC-3 cells. Schwann cells overexpressed TNFα mRNA (*P* < 0.01, Fig. 6b) and protein (*P* < 0.01, Fig. 6c) following co-culture with HSC-3 cells compared to control cells. The presence of precancer cells DOK increased mRNA (*P* < 0.05, Fig. 6b) expression but had no effect on protein levels of TNFα (Fig. 6c). DOK (*P* < 0.01) and HSC-3 cells (*P* < 0.001) also stimulated NGF release from Schwann cells (Fig. 6d). Adding recombinant TNFα to the culture media stimulated increased NGF release from Schwann cells (Fig. 6e).

### Nociceptive behaviors evoked by cancer-activated Schwann cells can be inhibited by TNFα inhibition

**Figure 7:**
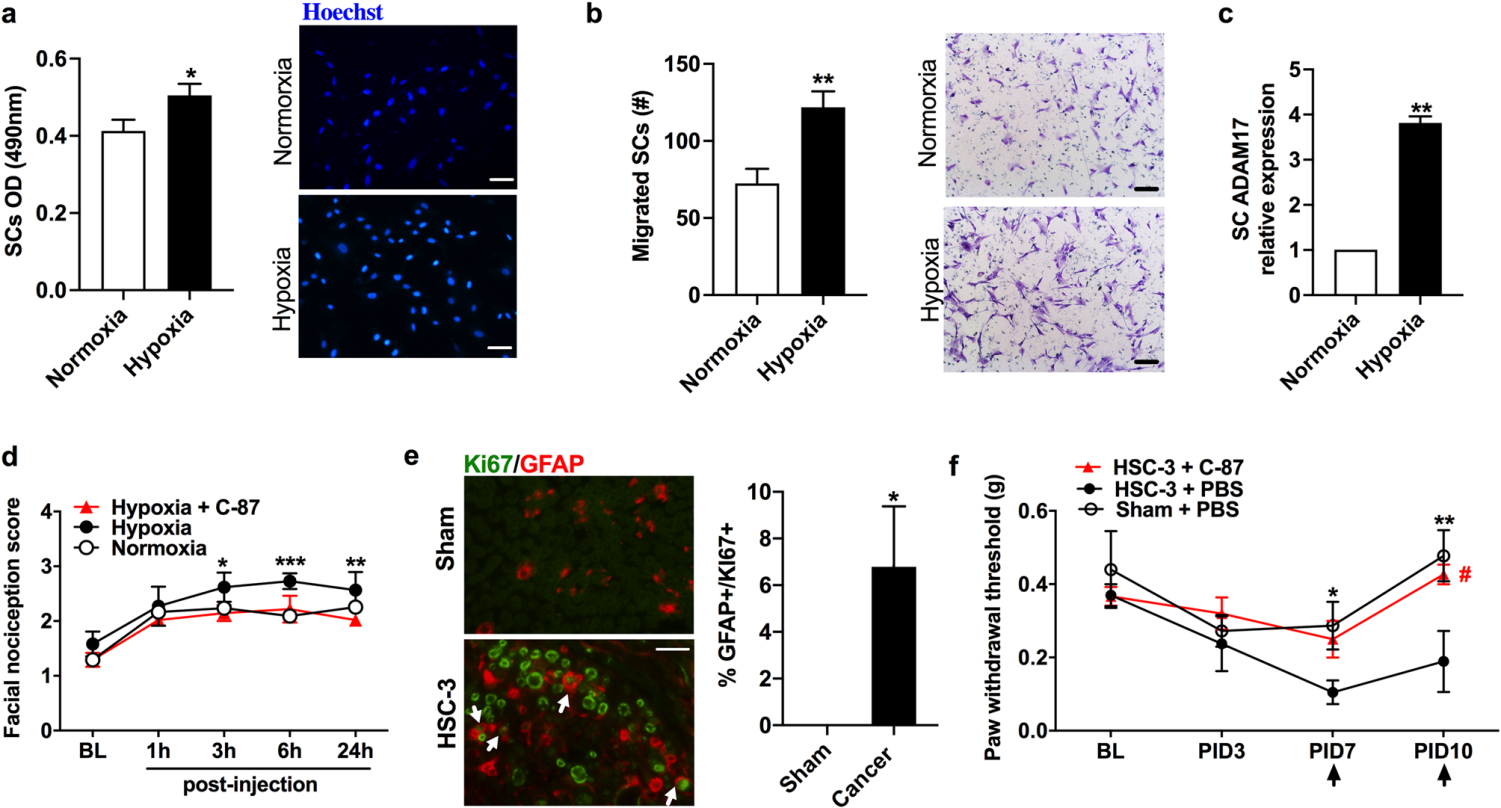
Activated Schwann cells induce nociceptive behaviors in mice. (**a**) Hypoxia increased Schwann cell proliferation compared to Schwann cells cultured under normoxic conditions. MTS assay. (**b**) Hypoxia increased Schwann cell migration towards 10% FBS compared to Schwann cells cultured under normoxic conditions. (**c**) Hypoxia significantly increased ADAM17 mRNA expression in Schwann cells compared to normoxia. (**a**–**c**) Student’s t test. (**d**) Facial nociception scores of mice at the baseline and at 1 h, 3 h, 6 h and 24 h following injection of supernatant from Schwann cells cultured under the following conditions: (1) normoxia, (2) hypoxia, and (3) hypoxia plus C-87 treatment. Mice who received hypoxic Schwann cell supernatant exhibited higher nociceptive scores compared to mice who received the normoxia supernatant or mice received hypoxia supernatant plus C-87 treatment. Two-way ANOVA. (**e**) Sciatic nerves with PNI exhibited increased Schwann cell proliferation (increased percentage of Ki-67 + /GFAP + cells) compared to nerve sections from the sham group. No Ki-67 positive cells were identified in the sham group. (**f**) Von Frey paw withdraw assay with the sciatic nerve PNI model. At day PID7 sham (PBS injection into the sciatic nerve) mice recovered from surgery/injection-induced pain whereas mice with tumor cells injected in the sciatic nerve continued to exhibit increased nociception. Paw withdrawal threshold was significantly lower in HSC-3 mice compared to sham mice at PID 7 and PID10. The SCC induced paw mechanical allodynia was reduced by C-87 treatment (arrows) at PID10 but not at PID7. Von Frey paw withdrawal assay was performed 1 h following C-87 injection. Images were taken using Nikon imaging software NIS-Elements F Ver4.60.00. Two-way ANOVA. *P < 0.05; **P < 0.01; ***P < 0.001.

Hypoxia is one of the main features of solid tumors and is known to activate Schwann cells^15^. We found that hypoxia (1% O_2_) induced increased Schwann cell proliferation (*P* < 0.05, Fig. 7a) and migration (*P* < 0.01, Fig. 7b) compared to Schwann cells cultured under normoxic conditions (18.6% O_2_). Hypoxia induced overexpression of ADAM17 (fourfold increase from the control, *P* < 0.01, Fig. 7c), a sheddase that is required to release soluble TNFα^39^. To test whether hypoxia-activated Schwann cells induce nociception mediated by TNFα, we injected mice into the tongue with supernatant obtained from Schwann cells cultured under three conditions: normoxia, hypoxia, and hypoxia with C-87 treatment. Facial von Frey was used to measure mechanical allodynia over time following injection. One hour after injection, all mice exhibited increased facial nociception from their respective baseline; no significant group difference was observed (Fig. 7d). Supernatant from Schwann cells cultured under hypoxic conditions induced increased facial allodynia compared to supernatant from Schwann cells cultured under normoxia condition at 3 (*P* < 0.05) and 6 h (*P* < 0.001) following injection. C-87 treatment (20 μM) in Schwann cell culture grown under hypoxic conditions reversed increased facial allodynia at 3 (*P* < 0.05), 6 (*P* < 0.001) and 24 h (*P* < 0.001) following injection (Fig. 7d). To examine the effect of Schwann cell activation on cancer pain in vivo, we used a sciatic nerve perineural invasion (PNI) model that is known to induce Schwann cell activation^13,40,41^. We used sham mice that received only the incision but no cancer implantation as a control. First, we examined whether PNI induced Schwann cell proliferation, a marker of activation. We used double immunofluorescence staining of Schwann cell marker, GFAP, and proliferation marker Ki-67 in sciatic nerve sections to identify and quantify proliferating Schwann cells. While no proliferating cells were detected in the sham nerve, we found a number of Schwann cells that were double positive for both GFAP and Ki67 in nerve sections from mice with PNI (Fig. 7e, P < 0.05); these results are consistent with several reports on other cancer types^13,14^. Next, we used hindpaw von Frey to measure mechanical allodynia in mice with sciatic nerve PNI or sham. C-87 treatment (12.5 mg/kg) was employed in a group of sciatic nerve PNI mice to determine a role for TNFα in PNI-mediated nociceptive behavior. All mice exhibited mechanical allodynia at PID3 (Fig. 7f). Sham mice exhibited increased mechanical thresholds at PID7 and recovered to their baseline mechanical thresholds at PID10. Tumor mice developed increased mechanical allodynia over time. Mice treated with C-87 at PID 7 and PID 10 had increased mechanical thresholds one hour following injection compared to vehicle (10% DMSO) injected tumor-bearing mice (*P* < *0.05,* Fig. 7f).

## Discussion

We provide clinical and preclinical evidence that TNFα promotes cancer progression and pain, at least in part through Schwann cell activation. TNFα is overexpressed in oral cancers and correlates positively with self-reported pain in patients. TNFα inhibition reduces oral cancer growth and cancer-induced nociceptive behavior. Oral cancer and TNFα can both induce Schwann cell activation. Using two murine models of oral cancer, we show that cancer-activated Schwann cells play a role in nociceptive behaviors in mice.

Our data support the role of TNFα in promoting oral cancer progression. TNFα overexpression has been reported in tumor tissues, blood, and saliva samples of oral cancer patients^42,43^; TNFα overexpression is associated with reduced overall survival and disease-free survival of oral cancer patients^30,33^. TNFα promotes oral cancer invasiveness and metastasis through autocrine signaling and paracrine signaling between cancer cells and cancer stromal cells in vitro^29,30,32,33^. TNFα recruits neutrophils to the environment that promotes cancer invadopodia formation and invasiveness^9,33^. In line with these findings, our data highlight a therapeutic potential of TNFα inhibitors for tumor reduction in vitro and in vivo. Adding C-87 directly into the oral cancer culture reduced cell proliferation, confirming the role of TNFα autocrine signaling in oral cancer proliferation. TNFα promotes oral cancer progression in part by regulating proinflammatory cytokines in the cancer microenvironment. In our animal models of oral cancer, C-87 reduced tumor size, along with the reduction of several proinflammatory markers/chemokines such as NGF, IL-1β, IL-33, and MIP3α that have been implicated in cancer proliferation, epithelial mesenchymal transition, transmigration, and extracellular matrix breakdown^8,44–48^.

We established a role of TNFα in oral cancer-induced chronic pain with several lines of evidence. We demonstrated a positive correlation between TNFα concentration in the tumor and self-reported pain in oral cancer patients. Previously, we have shown that TNFα is responsible for oral cancer supernatant-induced acute nociception^9^. While the supernatant injection model is valuable for studying the effect of oral cancer mediators on nociceptive behaviors, it does not reflect either the complexity of the cancer microenvironment or the chronic nature of cancer pain. TNFα can cause pain directly by activating and/or sensitizing primary afferent neurons^49^. TNFα can indirectly affect pain response by recruiting immune cells and regulate cytokine production within the tumor microenvironment^50,51^. Additionally, as our data suggest, TNFα can activate Schwann cells, leading to increased production of TNFα and NGF, further exacerbating pain.

Schwann cells are recognized as an emerging player in cancers of the skin, colon, prostate, and pancreas^13–16,18,52–54^. Schwann cells become activated as evidenced by dedifferentiating, proliferating, and migrating in the cancer microenvironment. Activated Schwann cells facilitate cancer metastasis and PNI, and modulate the immune system by interacting with cancer cells, neurons, immune cells, and other stromal cells in cancers of the skin, colon, prostate, and pancreas^13–16,18,52–54^. We found that in the presence of oral cancer, Schwann cells are activated with increased mobility and proliferation; activated Schwann cells chemo-attract oral cancer cells. TNFα inhibitor C-87 reduced Schwann cell activation and attraction between Schwann cells and oral cancer cells. The mutual attraction between Schwann cells and oral cancer cells can lead to cancer growth and invasion to the nerve (i.e., PNI)—a condition that is highly associated with increased locoregional recurrence, worse pain, and poor survival in patients^41,55–57^. In pancreas adenocarcinoma, precancerous pancreatic cells chemoattract Schwann cells, providing not only a path for dissemination of cancer cells to nerves, but also analgesia due to suppression of central glia by Schwann cell mediators^15,52^. Reduced Schwann cell activation is associated with increased pain in pancreatic cancer^15,52^. In contrast, Schwann cells are more activated in the presence of oral cancer cells than precancerous DOK cells, probably due to higher TNFα protein concentration in HSC-3 cells than DOK cells. In deed, HSC-3 cell but not DOK supernatant induces nociceptive response in mice^9,58^, and pain gets worse with disease progression in oral cancer patients^41,58,59^. It should be noted that DOK cells overexpressed TNFα mRNA. It is possible that DOK cells are not efficient in translating TNFα mRNA into proteins or they lack the sheddase ADAM17 to release soluble TNFα^39^.

The effect of cancer-activated Schwann cells on oral cancer pain is demonstrated in two animal models. In the first model we used hypoxia to induce Schwann cell activation. Cancer microenvironment is hypoxic; hypoxia is known to induce Schwann cell activation and cytokine release^14,52^. We showed that supernatant from hypoxia-activated Schwann cells induced increased mechanical hypersensitivity in mice. In the second model we inoculated oral cancer cells into the sciatic nerve to produce PNI and Schwann cell activation in vivo^40^. PNI is accompanied by Schwann cell activation in cancers of the pancreas and colon^13,14^. We showed that oral cancer invading to the sciatic nerve produced mechanical allodynia in mice. C-87 treatment reduced nociception induced by either hypoxia-activated Schwann cells or sciatic nerve PNI, suggesting a contributing role of TNFα in nociception in these two models. Both c-Jun and NF-kB have been postulated as immediate early genes that are critical for Schwann cell activation^60,61^. We report that in the setting of oral cancer, Schwann cells upregulate c-Jun. Blocking JNK that is upstream of c-Jun activation^61^ is also effective in pain relief in our mouse paw xenograft model. Cancer- or hypoxia-activated Schwann cells release nociceptive mediators such as IL-6, TNFα, CXCL2, and IL-8^11,15^; these mediators could sensitize primary afferent neurons to cause pain. Schwann cell activation causes myelin breakdown^41^; the loss of structural support and insulation by myelin sheath breakdown is another possible explanation of pain produced by activated Schwann cells.

The present study demonstrated that TNFα has a dual function in oral cancer progression and pain. Oral cancer- or TNFα-activated Schwann cells promote tumor progression and pain. Inhibition of TNFα or Schwann cell activation will provide potential treatments for oral cancer and associated pain.

## Methods

### Patients

The study was approved by the Institutional Review Board of New York University College of Dentistry. All patients provided written informed consent in accordance with the Declaration of Helsinki. All enrolled patients have biopsy-proven oral SCC with no history of prior surgical, chemotherapeutic, or radiation treatment. Oral cancer tissues and anatomically matched normal oral tissues were removed from patients during surgical treatment. Tissue samples were snap frozen and stored in liquid nitrogen. Since no instruments measure pain objectively, we asked patients to fill out a validated University of California San Francisco Oral Cancer Pain Questionnaire^20,21^ before surgery. The questionnaire consisted of eight questions on spontaneous and functional intensity, sharpness, aching and throbbing nature of the pain, which were rated on a visual analog scale (0–100 mm). None of the patients were taking analgesics or were receiving cancer treatment at the time of questionnaire completion. Total pain score is the sum of the scores from the 8 questions, each ranged from 0 to 100, with higher scores indicating more pain.

### Cell culture

Human oral SCC cells (HSC-3) (Japanese Collection of Research Bioresources, passage 4–10) were grown in DMEM (Invitrogen) containing antibiotic (penicillin/streptomycin, 10 U/ml) and 10% fetal bovine serum (FBS). DOK cells (Sigma Aldrich, passage 4–8) were cultured in DMEM with 10% FBS and 5 μg/ml hydrocortisone. Human primary cultures of Schwann cells (ScienCell Research Laboratories) were grown in Schwann Cell Media (SCM, ScienCell Research Laboratories). Cells were authenticated by ATCC and routinely tested for *Mycoplasma* (PlasmoTest Mycoplasma Detection Kit; InvivoGen).

### Animal models of SCC, nociceptive behavioral assays, and tumor size measurements

#### Animals

Six to eight-week-old female athymic NU/J (*Foxn1*^*nu*^) nude mice, BALB/cJ mice, and C57BL/6 mice were purchased from The Jackson Laboratory. Female mice were used as they exhibit stronger pain phenotype^62,63^ and male mice occasionally exhibit aggressive behaviors that require individual housing. Animal experiments were approved by the New York University Institutional Animal Care and Use Committee (IACUC) and performed in accordance with National Institutes of Health guide for the care and use of laboratory animals and the ARRIVE guidelines. Investigators blinded to drug treatment performed behavioral testing experiments.

#### 4NQO-induced oral cancer pain model and the gnawing-assay

C57BL/6 mice were ingested the carcinogen 4NQO (100 μg/ml; Sigma Aldrich, St. Louis, MO) in drinking water on an unrestricted basis for 16 weeks^9,58,62,63^. Fresh water was prepared with 4NQO stock solution (5 mg/ml in propylene glycol) weekly^9^. Control mice received water containing the equivalent dilution of propylene glycol alone. The dolognawmeter, a validated device and assay, was used to measure oral function and nociception^24^. Each mouse was placed into a confinement tube with two obstructing dowels in series; the mouse voluntarily gnaws through both dowels to escape the device. Each obstructing dowel is connected to a digital timer. The timer automatically stops when the mouse severs the dowel, recording the duration of time required to complete the behavior and escape the device. To acclimatize the mice and improve consistency in gnawing duration, all mice were trained for 5–7 sessions in the dolognawmeter. Training involves placing animals in the device and allowing them to gnaw through the obstructing dowels in exactly the same manner that they do during the subsequent experimental gnawing trials. A baseline gnaw-time was determined by the mean of the final three training sessions for each mouse. Following establishing a stable baseline gnaw-time, mice were treated with 4NQO for 16 weeks and the dolognawmeter assay was performed two times per week. Once 4NQO treated mice exhibited a significant increase of gnaw-time from baseline, they were randomized and received either a potent TNFα inhibitor C-87 (Sigma-Aldrich, 12.5 mg/kg, n = 8) or vehicle (10% DMSO in PBS, n = 5) via IP injection. In the propylene glycol treated mice, four received DMSO and six received C-87 treatment as control groups. C-87 is a small molecular TNFα antagonist developed by computer-aided drug design^22^. The dolognawmeter assay was performed one hour following the injection. Each mouse was normalized to its own baseline gnaw-time and data is presented as a percent change from the baseline. Following the last dolognawmeter assay, tongue tissues were harvested, fixed in 10% buffered formalin, and processed for paraffin embedding and slide preparation. Tissue sections were cut at 5 μm and stained with H&E to confirm the presence of the tumor. All mice included for analysis had pathology proven tongue cancer.

#### Paw cancer model and the paw withdrawal assay

NU/J mice are immunocompromised, which are permissive for the growth of human SCC. Nude mice were inoculated with 10^5^ HSC-3 cells in 50 μl of DMEM and Matrigel (1:1 by volume) into the plantar surface of the right hind paw^7,8^. Nociceptive behavior was measured using the von Frey paw withdrawal assay. Mice were allowed to acclimate to the behavior room, the experimenter, and the measuring device for 2 weeks before a baseline paw withdrawal threshold was taken. Animals were placed into individual Plexiglas boxes with meshed floor and were allowed to acclimate for 30 min. The mid-plantar right hind paw was stimulated with a series of von Frey fibers (bending forces: 0.02, 0.04, 0.07, 0.16, 0.4, 0.6, 1 and 2 g) with logarithmically incremental stiffness (TouchTest, North Coast Medical Inc.) using the “ascending stimulus” method^25,64^. The von Frey fibers were held perpendicular to the testing surface with sufficient force to cause buckling. A positive response was considered if the paw was sharply withdrawn and if there was an immediate flinching upon removal of the fiber. Three to six repetitive trials were averaged as the threshold for each mouse at different time points. By PID14, mice developed visible tumors in the paw and exhibited nociceptive behaviors measured by von Frey paw withdrawal assay. 100 μl vehicle (10% DMSO in PBS, n = 5), C-87 (12.5 mg/kg, n = 5), JNK inhibitor SP600125 (Sigma-Aldrich, 30 mg/kg, n = 5) was administered through IP injection starting on PID15. Paw withdrawal threshold was taken at 1, 3, 6, and 24 h (s) following treatment.

To analyze the effect of TNFα on tumor growth in vivo, nude mice were inoculated with 10^6^ HSC-3 cells in 50 μl of DMEM and Matrigel (1:1) into the plantar surface of the right hind paw. Every 3 days starting on PID 7 until PID 21, 100 μl vehicle (10% DMSO in PBS, n = 6) or C-87 (12.5 mg/kg, n = 7) was administered through IP injection. Paw volume was measured using a plethysmometer (IITC Life Science). On PID21, paws were harvested, fixed in 10% formalin, and processed for paraffin embedding and slide preparation. Tissue sections were cut at 5 μm and stained with H&E. Five sagittal sections from the paw midline that were 50 μm apart from each other were taken from each mouse and selected for relative tumor area quantification. A researcher trained by a board certified oral pathologist and blinded to experimental groups traced the tumor and paw area in the view field using the Nikon Eclipse TI microscope and Nikon imaging software NIS-Elements F Ver4.60.00, https://www.microscope.healthcare.nikon.com/products/software/nis-elements. Tumor area relative to paw area in H&E stained sections was calculated and compared between groups.

#### Schwann cell supernatant injection and the facial allodynia assay

When Schwann cells reached 90% confluence in the culture plate, the old media was replaced with 3 ml of low serum (1% FBS) SCM or 3 ml of low serum SCM with 20 μM of C-87. Schwann cells were then cultured under either normoxic or hypoxic (1% O_2_) conditions for 48 h before the supernatant was collected, centrifuged, and immediately utilized for cytokine quantification or injection. Schwann cell supernatant (40 µl) from the normoxic (n = 5), or hypoxic (n = 5), or hypoxic + C-87 (n = 5) culture condition was injected into the left cheek of BALB/cJ mice under isoflurane anesthesia. Facial von Frey testing was carried out according to a published methodology^65^. Mice were stimulated on their cheek with von Frey filaments ranging from 0.008 to 4 g force (11 filaments in total) in an ascending manner. The response score is reported as a numerical average of the 11 responses as they fit into the following response categories: 0, no response; 1, detection: mice turn their head slightly upon application of the filament to the face; 2, reaction: the mice turn the head away quickly, pull it backward or react as a single face wipe; 3, escape/ attack: the mice quickly escape from the filament or attack the filament by hand or by mouth, or wipe the face two times; 4, multiple face grooming: the mice respond to the filament simulation with more than 2 facial wipes continuously. Facial von Frey test was performed at the baseline, and at 1, 3, 6, and 24 h (s) after supernatant injection.

#### Sciatic nerve cancer perineural invasion (PNI) model

Athymic NU/J mice were anesthetized using isoflurane and their right sciatic nerve was exposed^41^. Oral cancer cells (1.5 × 10^4^ HSC-3 cells in 3 μl DMEM, n = 10) or PBS (sham, n = 5) were injected into the sciatic nerve, distal to the bifurcation of the tibial and common peroneal nerves. A formation of a bulb in the injection area indicates a good injection. After gently removing the needle, the nerves were then covered with the underlying muscles and the skin was closed with skin closure clips (Reflex 7). Mice were observed until fully recovered. Hind limb function was normal in all mice after the operation. 100 μl C-87 (12.5 mg/kg, n = 5) or vehicle (10% DMSO in PBS, n = 5) was administered through IP injection at PID7 and PID10 into cancer mice. Paw withdrawal threshold was collected using the von Frey filaments at baseline and 1 h following drug injections. On PID11, sciatic nerves were harvested, fixed in 10% buffered formalin, and processed for paraffin embedding. Tissue cross sections were cut at 5 μm thickness.

### Real-time PCR

Total RNA was isolated from Schwann cells using the Qiagen AllPrep DNA/RNA Micro Kit (Qiagen Inc.). Reverse transcription was carried out with Quantitect Reverse Transcription Kit (Qiagen Inc.) according to the manufacturer's instructions. Quantitative real-time PCR was performed with the Taqman Gene Expression Assay Kit (Applied Biosystems Inc.). We used the following primers: human ADAM Metallopeptidase Domain 17 (ADAM17, Hs01041915_m1), human TNFɑ (Hs00174128_ml), human ACTB (Hs99999903_m1) and human GUSB (Hs00939627_ml). The housekeeping genes β-actin (ACTB) and β-glucuronidase (GUSB) were used as the internal control gene. All primers were purchased from Life Technologies. Relative quantification analysis of gene expression data was calculated using the 2^−ΔΔCt^ method.

### Cell proliferation and migration assays

#### Real-time cell growth profiling

Real-time growth kinetics of HSC-3 cells was examined using the RTCA (xCELLigence System)^11,26,35^. Electrode impedance was represented as the Cell Index calculated with the manufacturer-developed algorithm. 1.0 × 10^4^ HSC-3 cells in 100 μl DMEM was added to each well. Cell growth was monitored for 18 h to reach the middle of the logarithmic growth phase. The plate was then removed from the RTCA apparatus, and 100 μl of freshly prepared media with different concentrations of C-87 or vehicle (0.2% DMSO) were added to each well. The plate was reinserted into the RTCA machine and cell growth was further assessed for up to 86 h. Six wells were used for each treatment. Normalization of the growth curves and slope calculations (based on the most linear phase of the entire growth curve) was performed using the RTCA Software Package 1.2. https://www.agilent.com/en/product/cell-analysis/real-time-cell-analysis/rtca-software/rtca-software-pro-741236.

#### CellTiter 96 AQ_ueous_ one solution cell proliferation assay

CellTiter 96 Aqueous One Solution (Promega) assay was performed following the manufacturer's instructions and previously reported^11^. Cells were seeded at a density of 1.0 × 10^4^ cells/well in 96-well plates. After 24 h of culture, cells were treated with 20 μM of C-87 and incubated for another 48 h. For the non-contact co-culture experiments, HSC-3 or DOK cells were added to cell inserts and co-cultured with Schwann cells for 48 h. As control samples, only cell culture media SCM was added to inserts. Optical density (O.D.) was measured at 490 nm using GloMax-Multi Microplate Multimode Reader (Promega). To visualize living cell density, in separate plates prepared in the same condition, the wells were washed to remove dead cells; attached living cells following co-culture were stained with Hoechst 33,342 (Thermo Fisher Scientific, 1:1000, 10 min incubation) and imaged under Nikon Eclipse TI microscope. Experiments were performed in triplicates.

#### Migration assays

Cell migration assays were performed using transwell Boyden chambers with an 8 µm pore size according to the manufacturer’s instructions (Corning) and our published protocols^11^. To examine Schwann cell migration towards HSC-3 cells, 1.0 × 10^4^ Schwann cells were seeded on the migration chambers; 1.0 × 10^4^ HSC-3 cells were seeded in the bottom chamber. Similarly, to examine HSC-3 cell migration towards Schwann cells, 1.0 × 10^4^ HSC-3 cells were seeded on the upper migration chambers; 1.0 × 10^4^ Schwann cells were seeded on the bottom chamber. C-87 (20 μM) and/or TNFα (20 ng/ml) were added to the bottom chamber. For the hypoxia experiments, Schwann cells were added to the top chamber and SCM containing 10% FBS was added to the bottom chamber. After 24 h incubation, the non-migrating cells were removed, and membranes containing migrated cells were fixed and stained with Diff-Quik (Microptic)^11^. The number of migrating cells on the lower side of the membrane was counted under a Nikon Eclipse TI microscope. Four photomicrographs per well were taken and quantified for data analysis. Experiments were performed in triplicates.

#### ELISA quantification

Frozen human oral cancer tongue tissues or cultured human Schwann cells were homogenized in ice-cold RIPA buffer containing 10% protease inhibitor cocktail. Human NGF and TNFα Quantikine ELISA kits were purchased from R&D systems. Total protein concentrations in each sample were quantified using a QuantiPro bicinchoninic acid (BCA) assay kit (Sigma-Aldrich). All samples were run in duplicate. The optical density was read at 450 nm wavelengths with the GloMax-Multi Microplate Multimode Reader (Promega).

#### Multiplex immunoassay

Pro-inflammatory cytokines were analyzed in tumors extracted from mouse paws using MILLIPLEX MAP magnetic bead immunoassay kits (EMD Millipore) as previously reported^9^. The kit contained 38 different cytokines/chemokines: TNFα, TNFβ, EGF, Eotaxin/CCL11, GCSF, GMCSF, IFNα2, IFNγ, IL1α, IL1β, IL1ra, IL2, IL3, IL4, IL5, IL6, IL7, IL8, IL10, IL12 (p40), IL12 (p70), IL13, IL15, IP10, IL17A, MCP1, MIP1α, MIP1β, VEGF, FGF2, TGFα, Flt3 ligand, Fractalkine, GRO, MCP3, MDC (CCL22), sCD40L, IL9. Mouse NGF was measured using a separate MILLIPLEX MAP magnetic bead kit. Mouse paws were homogenized with ice-cold RIPA buffer containing 10% protease inhibitor cocktail. The fluorescence intensity of magnetic beads was read on a Luminex 200 Instrument. The data were analyzed using MILLIPLEX Analyst 5.1 software, http://www.vigenetech.com/MILLIPLEXAnalystV51.htm. Each sample was run in duplicate. Six right paws from each group were used.

#### Immunofluorescence (IF) staining and western blot

For immunofluorescence staining Schwann cells were grown overnight at 37 °C with 20 ng/ml of recombinant human TNFα (R&D Systems) mixed in SCM. Control Schwann cells were cultured with inserts containing SCM alone. After 48 h of culturing, cells were washed, fixed in ice-cold methanol for 5 min, and permeabilized with 0.2% Triton X-100 for 5 min. Fixed cells or deparaffinized Sciatic nerve sections were incubated with Superblock (Thermo Fisher Scientific) for 1 h before addition of primary antibodies: mouse anti-c-Jun (1:50, Santa Cruz Biotechnology, sc-166540), rabbit anti-p75NTR (Alomone labs, ANT-007), mouse anti-MBP (1:50, Santa Cruz Biotechnology, sc-271524), rabbit anti-GFAP (1:100, Agilent, DAKO, GA52461-2), bovine anti-GFAP Alexa Fluor 594 (1:500, Santa Cruz Biotechnology, sc-33673 AF594), and rabbit anti-Ki-67 (1:500, Thermo Fisher Scientific, MA5-14520) at 4 °C overnight. After 3 washes with PBS, the coverslips were incubated with anti-rabbit Alexa Fluor 594 (1:500, Thermo Fisher Scientific) or rabbit anti-mouse Alexa-Fluor 488 (1:500, Thermo Fisher Scientific), goat anti-rabbit Alexa-Fluor 488 (1:500, Thermo Fisher Scientific) in PBS for 1 h at room temperature. Cover slips were washed and mounted on slides in aqueous mounting medium with DAPI to stain the nuclei (Santa Cruz Biotechnology) and imaged with a Nikon Eclipse TI microscope. Five images were taken for each coverslip. Fluorescence intensity of each image was quantified using Nikon imaging software NIS-Elements F Ver4.60.00. Experiments were run in triplicate.

For western blot analysis, Schwann cells were cultured in SCM mixed with 20 ng/ml of recombinant human TNFα or vehicle or with inserts containing HSC-3, DOK, or SCM alone (non-contact co-culture) for 48 h before harvest. Protein extraction and quantification were conducted using established protocols^11^. Cells were lysed and homogenized in ice-cold RIPA buffer (Thermo Fisher Scientific) with 10% protease inhibitor cocktail (Thermo Fisher Scientific). Homogenates were centrifuged at 13,000 g for 10 min at 4 °C. The supernatant was collected and protein concentration was determined using the bicinchoninic acid (BCA) protein assay kit (Thermo Fisher Scientific). 20 μg of protein extract was fractionated on a 12% Mini-Protean TGX gel (Bio-Rad) and transferred onto nitrocellulose membranes (Thermo Fisher Scientific). Membranes were blocked for 1 h with 5% non-fat milk in PBS containing 0.1% Tween-20, and then incubated overnight at 4 °C with the following antibodies: rabbit anti-GFAP (1:500, Agilent, DAKO, GA52461-2), mouse anti-c-Jun (1:100, Santa Cruz Biotechnology, sc-166540), rabbit anti-GAPDH antibody (1:1000, Cell Signaling, 2118), mouse anti-p75 (1:100, Santa Cruz Biotechnology, sc-271708), and mouse anti-MBP (1:100, Santa Cruz Biotechnology, sc-271524). HRP-conjugated goat anti-rabbit IgG (Santa Cruz Biotechnology, sc-2030) or goat *anti*-*Mouse* IgG (Thermo Fisher Scientific, 62–6520) were used as secondary antibodies at a 1:2500 dilution. The signal was detected by Clarity Western ECL Substrate (Bio-Rad) and analyzed using ChemiDoc MP Imaging System with Image Lab Software 6.1, https://www.bio-rad.com/en-us/product/image-lab-software?ID=KRE6P5E8Z.

### Statistical analysis

We used Prism 6.0 h statistics software package (https://www.graphpad.com/support/prism-6-updates/) for all data analysis. Student's *t*-test or Mann–Whitney U test was used for two-group analysis. One-way ANOVA or Kruskal–Wallis test with Dunnett’s post hoc analysis were used to compare multiple groups. Two-way ANOVA with one within-subject factor (time) and one between-subject factor (treatment) followed by Holm-Sidak posthoc tests was used to compare the effect of different treatments over time. Correlation between TNFα and pain scores in patients was determined using the Pearson correlation coefficient. *P* < 0.05 was considered statistically significant. Results were presented as mean ± standard error of the mean (SEM).

## Supplementary information

Supplementary Information.

## Data Availability

The datasets generated and/or analyzed during the current study are available from the corresponding author on reasonable request.
