# Supplementary material for: TNFα promotes oral cancer growth, pain, and Schwann cell activation
Source: Sci Rep. 2021 Jan 19;11:1840. doi: 10.1038/s41598-021-81500-4 (PMC7815837; doi:10.1038/s41598-021-81500-4)

TNFα promotes oral cancer growth, pain, and Schwann cell activation

Elizabeth Salvo[1,2,3], Nguyen H. Tu[1,2,3], Nicole N. Scheff[4], Zinaida A. Dubeykovskaya[1,2,3], Shruti A. Chavan[5], Bradley E. Aouizerat[1,2], Yi Ye[1,2,3]*

[1]Bluestone Center for Clinical Research, New York University College of Dentistry

[2]Department of Oral Maxillofacial Surgery, New York University College of Dentistry

[3]Department of Molecular Pathobiology, New York University College of Dentistry

[4]Department of Neurobiology, School of Medicine, University of Pittsburgh, Pittsburgh, PA, USA

[5]Graduate School of Arts & Science, Department of Biology, New York University

S1. Gel-blots for Figure 5.

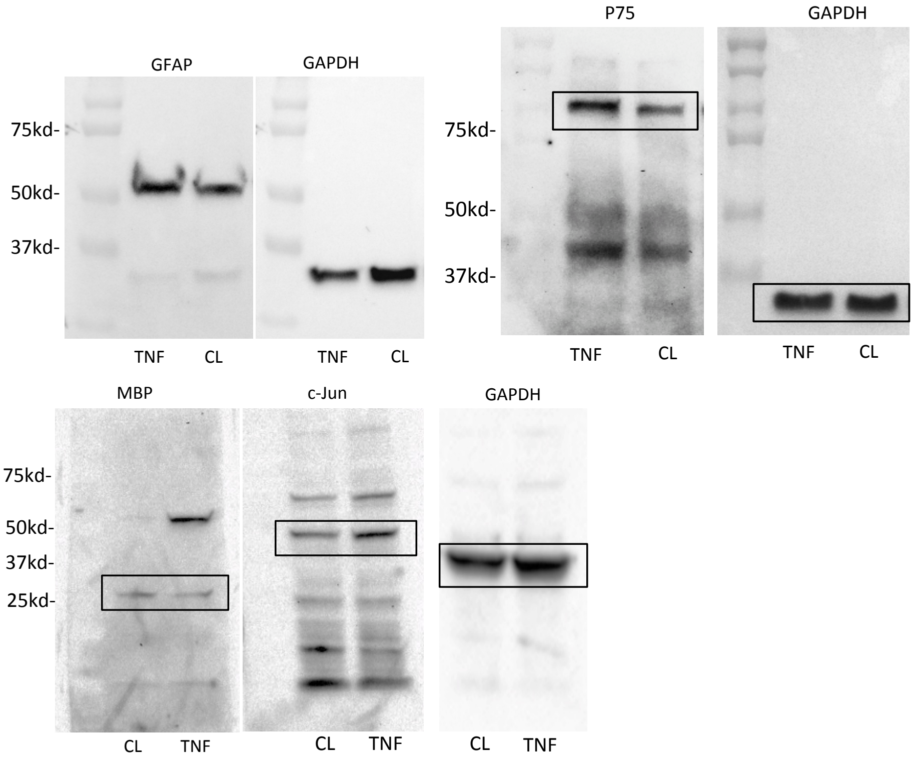

S2. Gel blots for Figure 6.

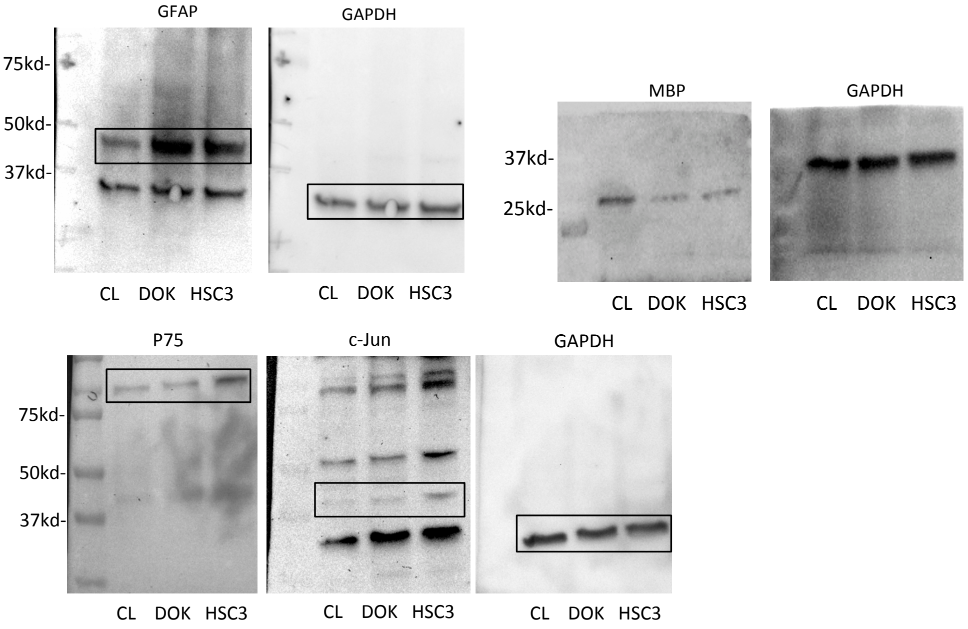

Supplement: Supplementary file 1 — Supplementary Information. [file 41598_2021_81500_MOESM1_ESM.pdf]
